# Quantitative FRET Imaging to Visualize the Invasiveness of Live Breast Cancer Cells

**DOI:** 10.1371/journal.pone.0058569

**Published:** 2013-03-13

**Authors:** Shaoying Lu, Yi Wang, He Huang, Yijia Pan, Eric J. Chaney, Stephen A. Boppart, Howard Ozer, Alex Y. Strongin, Yingxiao Wang

**Affiliations:** 1 Department of Bioengineering, University of Illinois at Urbana-Champaign, Urbana, Illinois, United States of America; 2 Neuroscience Program, University of Illinois at Urbana-Champaign, Urbana, Illinois, United States of America; 3 Beckman Institute for Advanced Science and Technology, University of Illinois at Urbana-Champaign, Urbana, Illinois, United States of America; 4 Department of Chemical Engineering, Center of Biophysics and Computational Biology, Department of Molecular and Integrative Physiology, University of Illinois at Urbana-Champaign, Urbana, Illinois, United States of America; 5 Cancer Center and Department of Medicine, University of Illinois, Chicago, Illinois, United States of America; 6 Sanford Burnham Medical Research Institute, La Jolla, California, United States of America; 7 Department of Bioengineering, University of California San Diego, La Jolla, California, United States of America; Duke University, United States Of America

## Abstract

Matrix metalloproteinases (MMPs) remodel tumor microenvironment and promote cancer metastasis. Among the MMP family proteases, the proteolytic activity of the pro-tumorigenic and pro-metastatic membrane-type 1 (MT1)-MMP constitutes a promising and targetable biomarker of aggressive cancer tumors. In this study, we systematically developed and characterized several highly sensitive and specific biosensors based on fluorescence resonant energy transfer (FRET), for visualizing MT1-MMP activity in live cells. The sensitivity of the AHLR-MT1-MMP biosensor was the highest and five times that of a reported version. Hence, the AHLR biosensor was employed to quantitatively profile the MT1-MMP activity in multiple breast cancer cell lines, and to visualize the spatiotemporal MT1-MMP activity simultaneously with the underlying collagen matrix at the single cell level. We detected a significantly higher level of MT1-MMP activity in invasive cancer cells than those in benign or non-invasive cells. Our results further show that the high MT1-MMP activity was stimulated by the adhesion of invasive cancer cells onto the extracellular matrix, which is precisely correlated with the cell’s ability to degrade the collagen matrix. Thus, we systematically optimized a FRET-based biosensor, which provides a powerful tool to detect the pro-invasive MT1-MMP activity at single cell levels. This readout can be applied to profile the invasiveness of single cells from clinical samples, and to serve as an indicator for screening anti-cancer inhibitors.

## Introduction

Matrix metalloproteinases (MMPs) are a family of zinc-dependent proteases, which play important roles in tumor metastasis, inflammation, and development [1], [2]. The human MMP family contains 23 members: 16 secreted and 7 membrane-anchored proteases [1]. The majority of MMPs are pro-tumorigenic in cancer, although certain individual MMPs, such as MMP-8, can assume an opposite protective role [3]–[5]. Broad-spectrum inhibition of MMP has been shown to be beneficial in clinical trials and animal models if started at an early stage, but not at an advanced stage, of tumor progression [6]. Therefore, it is essential to investigate specific roles played by individual MMPs at the cellular level, as well as to evaluate the effectiveness of specific MMP inhibitors, for the purpose of developing anti-MMP cancer therapy.

Among the MMP family proteases, membrane type-1 MMP (MT1-MMP; also called MMP-14) is essential for the peri-cellular proteolysis of collagen matrix in the basement membrane, as well as tumor cell invasion and metastasis [7]-[11]. MT1-MMP can also activate the MMP-2 proenzyme [2] and regulate multiple adhesion receptors to promote invasion [12]. MT1-MMP is synthesized in an inactive form that consists of an auto-inhibitory prodomain, a zinc-binding catalytic domain, a hemopexin-like domain and a transmembrane domain followed by a short cytoplasmic tail [13], [14]. Furin-like convertases and MMP-dependent proteolysis can cause the removal of the prodomain and the subsequent activation of MT1-MMP [15]–[17]. The activated MT1-MMP can then localize at the cellular surface, internalized via the clathrin-dependent pathway [18], and recycled back to the plasma membrane via exocytosis [19].

Consistent with its tumorigenic function, MT1-MMP is widely expressed in multiple cancer types, and its proteolytic activity is linked to multiple invasive cancer forms [20]–[25]. As a result, the measurement of cellular MT1-MMP activity can provide an indicator of aggressive cancer types, as well as a foundation of the treatment selection for cancer patients. However, our ability to quantitatively and selectively record the MT1-MMP activity at the single cell level is still limited. Therefore, it is imperative to develop highly sensitive and specific MT1-MMP biosensors as molecular tools for cancer research and cancer therapeutics [26].

FRET-based molecular beacons and probes have recently been developed to detect MT1-MMP cleavage activity in medium and extracellular matrix, respectively [27], [28]. However, these fluorescence probes were cleaved more efficient by MMP-9 than MT1-MMP and lacked subcellular resolution. Our group previously developed a cleavage-activated MT1-MMP FRET biosensor (namely the NL biosensor) that contains a cleavable NL sequence (CPKESC*NL*FVLKD) from the MMP-2 pro-enzyme [29]. The application of this original NL biosensor showed that MT1-MMP was predominantly activated at the migration front of live cells, as confirmed by others [28]. The NL biosensor, however, exhibited a slow response and limited sensitivity toward MT1-MMP cleavage, which warranted further improvement.

Two strategies have recently become available for improving the sensitivity and specificity of this biosensor. First, by MMP proteolysis, we identified several efficient substrate sequences, which can be used to replace the NL sequence in the biosensor [30], [31]. Second, it has been reported that additional linkers flanking the substrate sequence can generally enhance the sensitivity of FRET biosensors [32]. In this paper, we systematically implemented these modifications on the NL biosensor, and quantitatively characterized both the sensitivity and the specificity of several novel and significantly improved MT1-MMP biosensors. We then selected the most sensitive AHLR biosensor, which exhibited both the optimized cleavage sequence and linker length, to record the MT1-MMP activity in multiple breast cancer cell lines. Our results indicate that the invasiveness of the cells is correlated with the level of the cellular MT1-MMP activity, which can be accurately detected and profiled by the AHLR biosensor.

## Results

### *In vitro* optimization of the sensitivity of the MT1-MMP biosensor

An engineered biosensor contains an enhanced cyan fluorescence protein (ECFP) as the FRET donor, a substrate peptide with or without linker sequences, and a yellow fluorescence protein (YPet) as the FRET acceptor (Fig. S1A). At the rest state, there is strong energy transfer between ECFP and YPet. When the biosensor is cleaved by the catalytically active MT1-MMP, ECFP separates from YPet, causing a decrease of energy transfer, or an increase of ECFP/FRET emission ratio (Fig. S1B). Thus, the ECFP/FRET ratio signal of the biosensors can be used to represent the recorded MT1-MMP activity.

**Figure 1:**
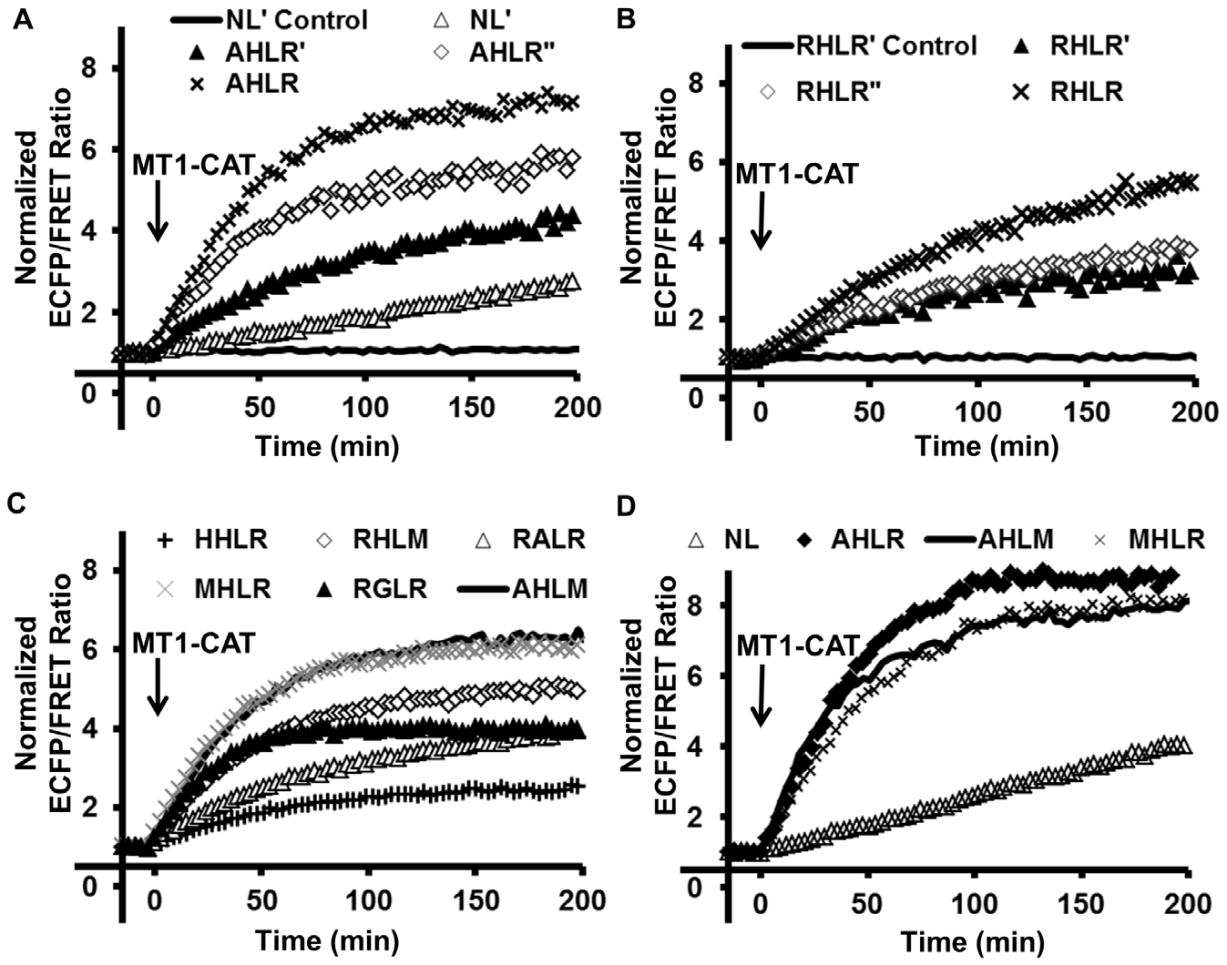
*In vitro* characterization of the sensitivity of MT1-MMP biosensors. **(A)** Comparing the responses of different MT1-MMP biosensors upon the incubation with the catalytic domain of MT1-MMP (MT1-CAT). The substrate peptides are NL only (NL’), AHLR only (AHLR’), AHLR flanked by GGS linkers (AHLR”) or by GGSGGT linkers (AHLR). The label “NL’ control” indicates no MT1-CAT incubation. **(B)** Characterization of the biosensors with substrate peptides RHLR only (RHLR’), and RHLR flanked by GGS (RHLR”) or GGSGGT linkers (RHLR). The label “RHLR’ control” indicates no MT1-CAT incubation. **(C)** Characterization of the MT1-MMP biosensors containing different substrate peptides with GGSGGT linkers (HHLR, RHLM, RALR, MHLR, RGLR, or AHLR). **(D)** Comparison of the MT1-MMP biosensors containing the GGSGGT linker and different substrate peptide sequences (NL, AHLR, AHLM or MHLR).

To examine the effect of linkers on the biosensor sensitivity, we engineered FRET biosensors with a promising substrate sequence (AHLR or RHLR) flanked by flexible linkers of either the GGS or GGSGGT sequence (Fig. S1A)[32]. The AHLR and RHLR sequences are two most sensitive and specific MT1-MMP substrate sequences among the eight candidates, which were predicted by the substrate phage library cleavage assay to be significantly more efficient than the original NL sequence (Table S1) [29]. When the purified biosensors were co-incubated with the catalytic domain of MT1-MMP (MT1-CAT) to allow cleavage, the ECFP/FRET ratio of the biosensor with the AHLR sequence flanked by the GGSGGT linker (named the AHLR biosensor) increased 7-fold, with a response 38% and 109% higher than the biosensor with the GGS linker[30] and that without the linker (AHLR sequence alone), respectively (Fig. 1A). The response of the AHLR biosensor with the GGSGGT linker to MT1-CAT cleavage is also 5 times of the original NL biosensor[29]. Similarly, the RHLR biosensor with the GGSGGT linker was superior when compared to the RHLR biosensors with the GGS linker or without the linker (RHLR sequence alone) (Fig. 1B). These results indicate that the flexible linkers GGSGGT can improve the sensitivity of MT1-MMP biosensors, possibly by allowing the active MT1-CAT more space to access and cleave the substrate sequence. Therefore, all the biosensor constructs in the remaining manuscript were engineered with the GGSGGT linker. We refer to the biosensors by the names of their substrate sequences.

We next examined the influence of different substrate sequences on the biosensors sensitivity. Among the other six promising peptide sequences examined, including HHLR, RHLM, RALR, MHLR, RGLR and AHLM, the biosensor with the AHLM or MHLR sequence was found to be the most sensitive to MT1-CAT cleavage *in vitro,* with a 6-fold increase in the ECFP/FRET ratio (Fig. 1C). We then compared side-by-side the three most sensitive biosensors, with the AHLR, AHLM, or MHLR sequence flanked by the GGSGGT linker (Figs. S2A and 1D). As shown in Figure 1D, the AHLR biosensor was the most sensitive among all biosensors examined.

### *In vitro* examination of the specificity of the MT1-MMP biosensors

**Figure 2:**
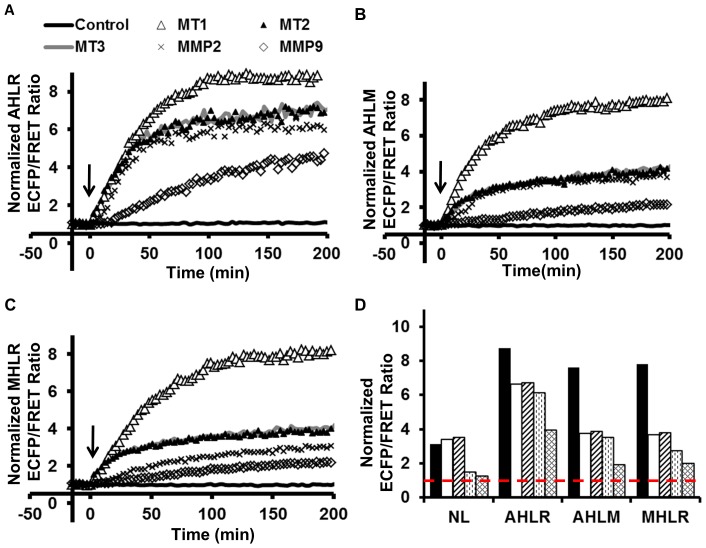
Characterize the specificity of the three most sensitive MT1-MMP biosensors. The time courses of the ECFP/FRET emission ratio of the **(A)** AHLR, **(B)** AHLM, **(C)** MHLR biosensors upon the cleavage by different active catalytic domains from MT1-, MT2-, MT3-MMP, or the full length active MMP2 or MMP9. **(D)** The quantified maximal ECFP/FRET ratio after cleavage is compared among the NL-, AHLR-, AHLM-, and MHLR biosensors with different MMP catalytic cleavage. The values were calculated by averaging the response at 2-2.5 hrs after MT1-CAT incubation.

To assess the biosensor specificity, purified AHLR, AHLM and MHLR biosensors were cleaved *in vitro* using different catalytic domains of MT1-MMP, MT2-MMP, and MT3-MMP, and full length MMP-2 and MMP-9. As shown in Figure 2, all three biosensors favored MT1-MMP cleavage, as predicted by our phage library cleavage assay [31]. Therefore, the AHLR biosensor was chosen as a specific and the most sensitive toward MT1-MMP proteolysis, although the AHLM and MHLR biosensors were moderately more specific (Fig. 2D).

Furthermore, we evaluated the selectivity of the AHLR biosensor against MMP-8. The results showed that the AHLR biosensor was resistant to MMP-8 proteolysis (Fig. S3). This selectivity is important since, in contrast to MT1-MMP, MMP-8 displayed an anti-tumorigenic property for certain cancer types [4], [5]. Therefore, the AHLR biosensor reports the pro-invasive activity of MT1-MMP but not the protective activity of MMP-8 and, thus, can be applied to profile the invasiveness of cancer cells.

### Profiling the cellular MT1-MMP activity in live cancer cells using the AHLR biosensor

We engineered the AHLR biosensor to express at the extracellular surface of the plasma membrane, where active MT1-MMP functions (Fig. S2B) [18], [29]. Similar to the *in vitro* assay, the MT1-MMP cleavage can allow the acceptor to diffuse away from the donor at the cellular membrane, causing a decrease of energy transfer and an increase of the ECFP/FRET emission ratio. Therefore, the ECFP/FRET ratio can be applied to quantify the cellular MT1-MMP activity.

**Figure 3:**
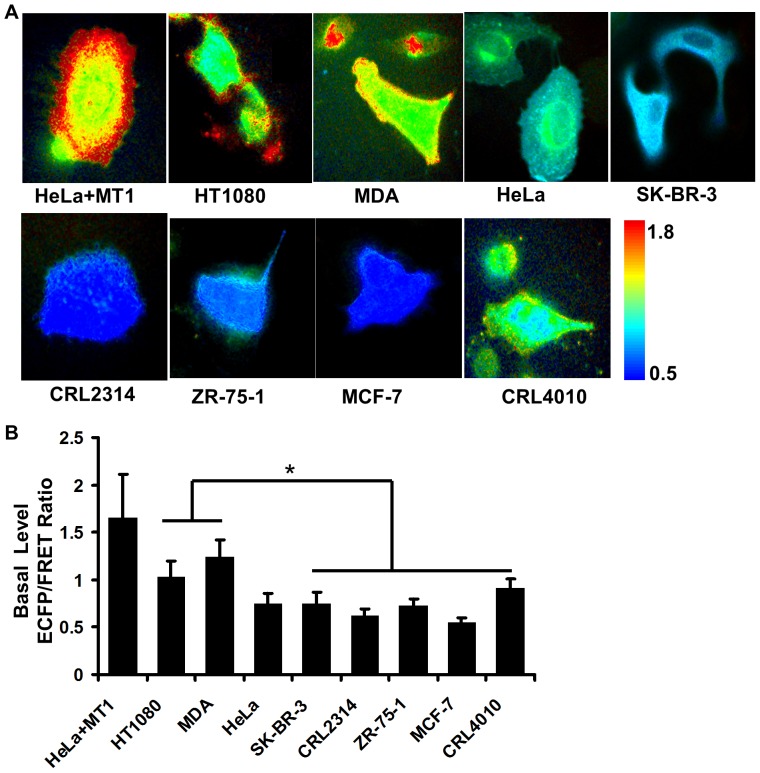
Profile the basal level of apparent MT1-MMP activity in cancer cell lines. **(A)** The ECFP/FRET ratio images of representative cancer cells with the AHLR biosensor; **(B)** the quantified ECFP/FRET ratio values of the cancer cell lines (MEAN±SEM). (*) indicates statistically significant difference by the two-tail t-test with unequal variance. For example, between MDA-MB-231 (n = 26) and CRL4010 (n = 21) cells, p-value = 5.0e-10. Between HT1080 (n = 33) and CRL4010 (n = 21) cells, p-value = 9.5e-4.

The examined cell lines had different MT1-MMP expression levels and invasive capabilities. These cells include the highly invasive HT1080 fibrosarcoma and MDA-MB-231 breast carcinoma cells (both with high levels of MT1-MMP), non-invasive SK-BR-3, CRL2314, ZR-75-1 and MCF-7 breast cancer cells, and CRL4010 immortalized epithelial cells. In addition, HeLa cells with a minimal level of endogenous MT1-MMP, and their counter parts with overexpressed exogenous MT1-MMP, were used as negative and positive controls, respectively (Table S2). The ECFP/FRET ratio images of the AHLR biosensor in cells cultured on cover glass were quantified and averaged at the peripheral regions where active MT1-MMP has been shown to concentrate [29] (Fig. 3A). The quantified results revealed that the MT1-MMP activity was significantly higher in the invasive HT1080 and MDA-MB-231 cells than other cell lines. Specifically, the MT1-MMP activity was low in the non-invasive SK-BR-3, CRL2314, ZR-75-1, MCF-7, and CRL4010 cells (Figs. 3A–B). These results suggest that the AHLR biosensor can be applied to assay the MT1-MMP activity in individual cells with various levels of invasive potential.

**Figure 4:**
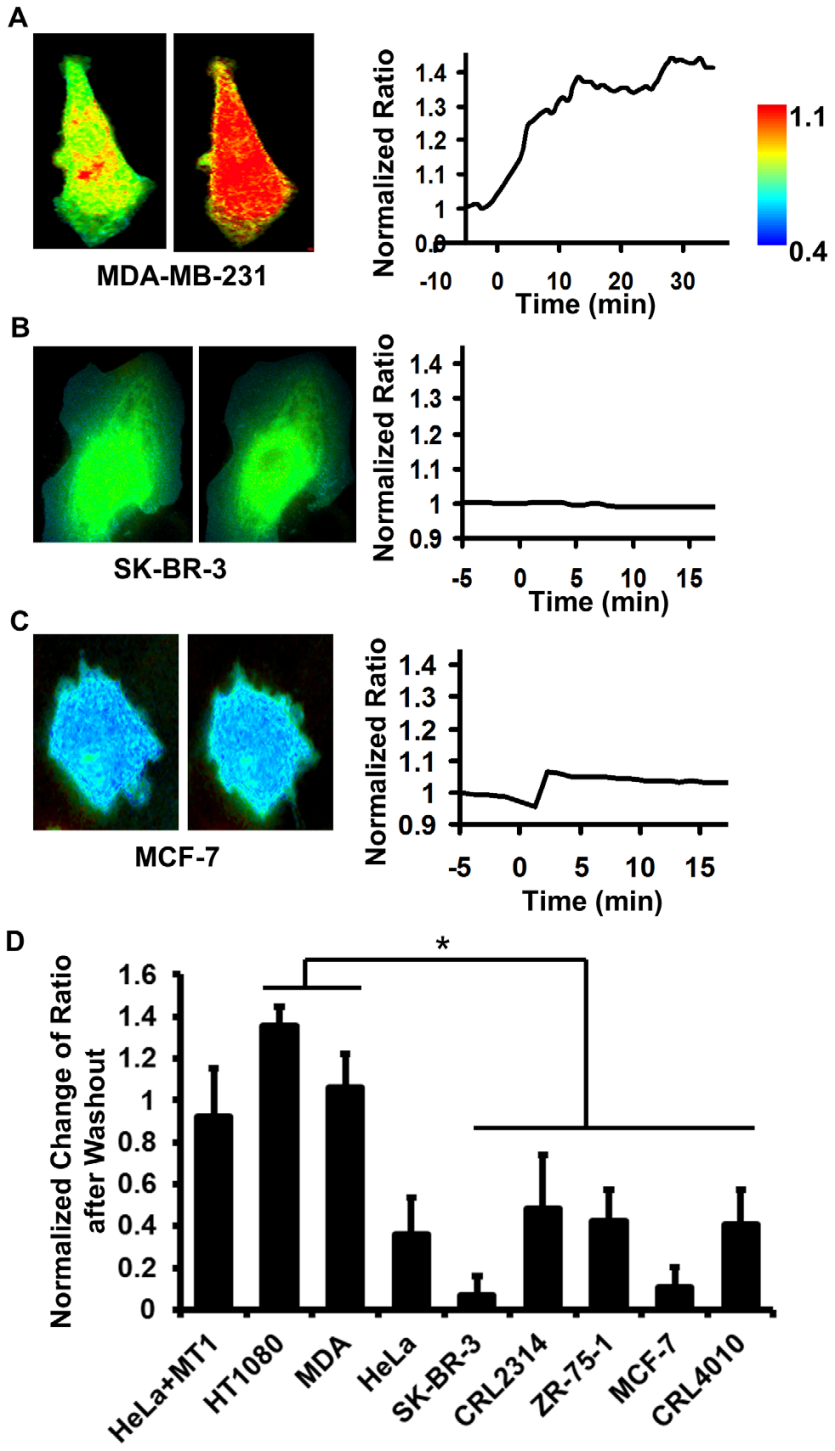
Profile the invasive MT1-MMP activity in breast cancer cell lines utilizing GM6001 washout assay. For breast cancer cell lines including **(A)** MDA-MB-231, **(B)** SK-BR-3, and **(C)** MCF-7, the ECFP/FRET emission ratio images are shown for a representative cell before (left) and after (middle) GM6001 washout. The time course of the quantified ECFP/FRET ratio value normalized to its average value before GM6001 washout is plotted in the right panel. Another representative MDA-MB-231 cell under the GM6001-washout assay is shown in Movie S1. **(D)** The quantified values of the increase in ECFP/FRET ratio after GM6001 washout are compared among different cell lines (MEAN±SEM). (*) indicates statistically significant difference by two-tailed t-test with unequal variance, p-value<0.01 between MDA-MB-231 (n = 10) and CRL2314 (n = 15) cells.

To further eliminate the contribution of cellular proteinases other than MMPs in cell assays of the biosensor cleavage, the cells with the membrane-bound AHLR biosensor were pretreated with GM6001, a potent broad-range hydroxamate inhibitor of MMPs [33], [34]. The ECFP/FRET ratio signal observed in the presence of GM6001 represents the background of the biosensor cleavage by non-MMP proteinases. GM6001 was then washed out to allow the cleavage of the biosensor by the cellular MT1-MMP. The increase of the ratio after GM6001 wash-out was then quantified at cell peripheral regions to determine the biosensor cleavage by MT1-MMP in different cell types (Figs. 4A–C and S4). The results were consistent with our basal-level cell profiling studies but with better sensitivity in differentiating highly invasive cancer cells from others. For example, invasive MDA-MB-231 and HT1080 cells exhibited a much higher level of the relatively MT1-MMP-specific activity in the GM6001-washout assay than non-invasive SK-BR-3 and MCF-7 cells (Fig. 4D and Movie S1).

### Adhesion to fibronectin up-regulates cellular MT1-MMP activity

**Figure 5:**
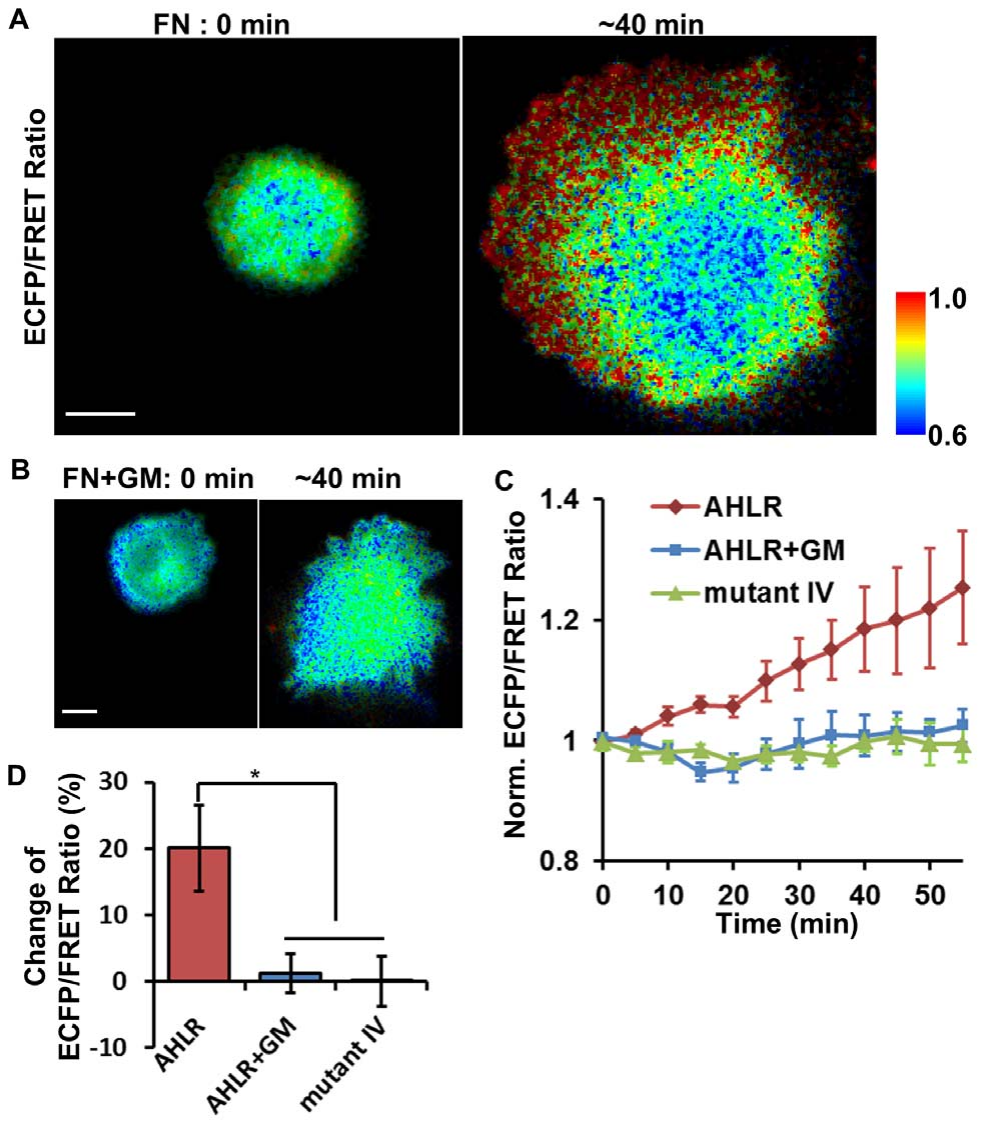
The MT1-MMP activity was up-regulated by cell adhesion to matrix in MDA-MB-231 cells. **(A-B)** The ECFP/FRET ratio images of a representative cell expressing the AHLR biosensor are shown at 0 min (left) and 40 min (right) after adhesion to fibronectin-coated cover glass with (B) or without (A) the pretreatment of GM6001. The same cell in panel (A) is shown in Movie S2. **(C)** The quantified time courses of ECFP/FRET ratio value of the MDA-MB-231 cells expressing the AHLR biosensor with or without GM6001 treatment, and that with the IV negative mutant biosensor during the adhesion process on fibronectin. **(D)** The averaged ECFP/FRET ratio increase of the cells in (C) 20–30 min after adhesion (MEAN±SEM). Scale bars: 10 µm. (*) indicates statistically significant difference with p-value<0.01.

Because the MT1-MMP activity is essential for efficient cell invasion and migration through the matrix, we hypothesized that high MT1-MMP activity observed in invasive cancer cells were associated with cell-matrix interaction. As a result, screening cancer cells in adhesion may provide a more accurate measurement than the screening methods targeting cells in suspension. To test this hypothesis, the MDA-MB-231 cells were transfected with the AHLR-biosensor and then dropped onto the cover glass coated with fibronectin, an extracellular matrix protein. The ECFP/FRET ratio images were monitored during this dynamic adhesion process. During 40–50 min following adhesion, the ECFP/FRET ratio increased significantly and was unevenly distributed at subcellular levels, with a high ratio at the active lamellipodia region (Fig. 5A and Movie S2). This increase of ECFP/FRET ratio and the subcellular difference was completely suppressed by GM6001 (Fig. 5B–D). As control, there was no increase of the ECFP/FRET ratio in cells transfected with a similar biosensor containing a substrate sequence not cleavable by MT1-MMP (substrate sequence: CPKESC*IV*FVLKD, Fig. 5C and D) [29]. These results suggest that adhesion to fibronectin can enhance the level of active MT1-MMP in invasive MDA-MB-231 cells. Therefore, upon adhesion on extracellular matrix, invasive cancer cells can display a higher MT1-MMP activity than those in suspension, indicating that adhesive cells can provide a more sensitive readout of cellular MT1-MMP activity.

### MT1-MMP activity correlates with collagen degradation in single live cells

**Figure 6:**
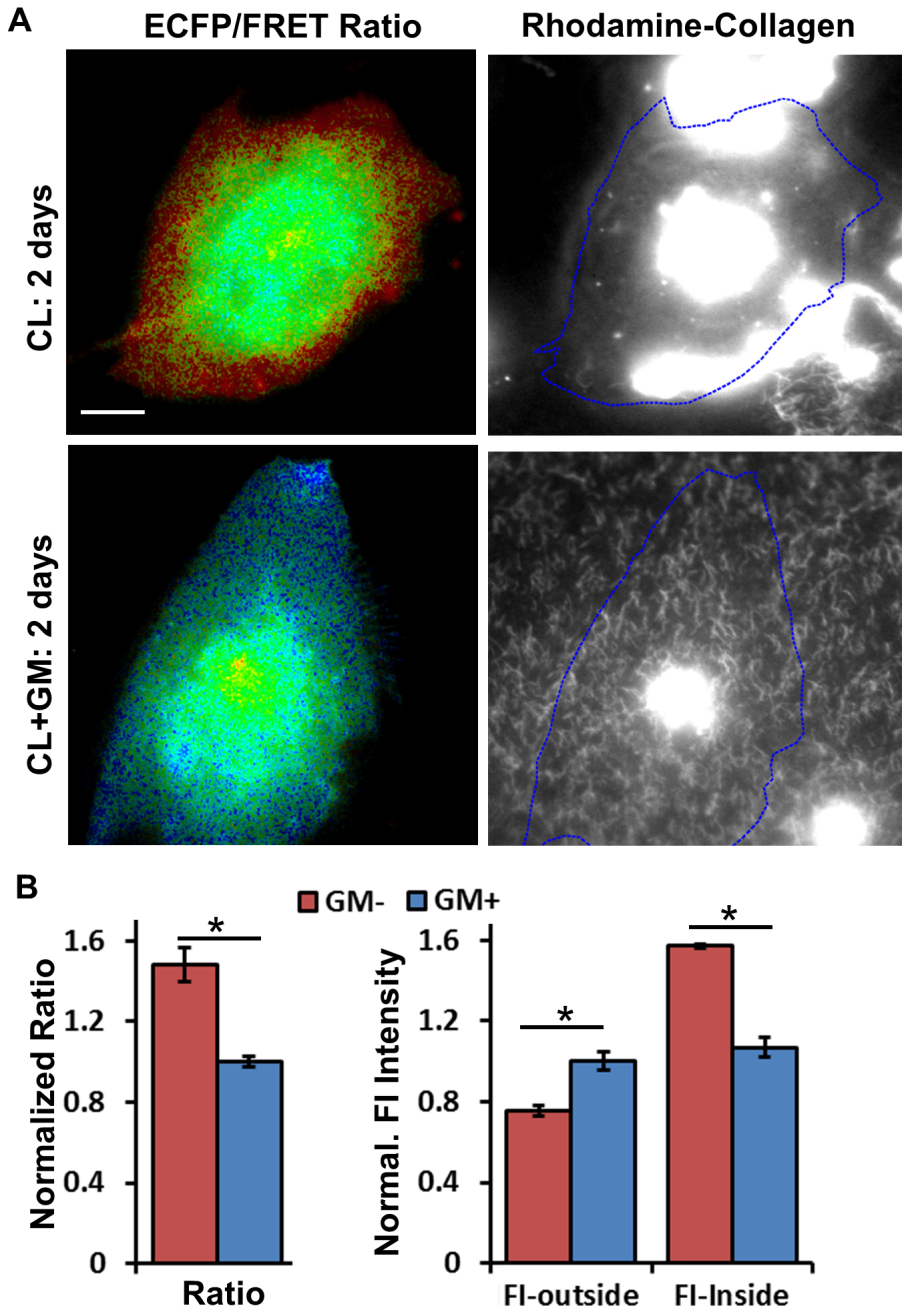
The MT1-MMP activity in MDA-MB-231 cells is correlated with the invasiveness of the cells on cross-linked collagen films. (A) The ECFP/FRET ratio and rhodamine-collagen intensity images of a representative cell expressing AHLR-MMP biosensor 2 days after adhesion to collagen (CL)-coated cover glass, with (top) or without (bottom) GM6001 inhibition. The shape of the cells is outlined in blue dashed lines. Scale bars: 10 µm. (B) Left panel: The quantified ECFP/FRET ratio (Ratio), and right panel: the fluorescence intensity of rhodamine collagen outside (FI-outside) and inside (FI-inside) the cell body were compared between cells pretreated with (GM+) or without (GM-) GM6001 inhibition (MEAN±SEM). (*) indicates statistically significant difference with p-value<0.01.

Because MT1-MMP efficiently degrades type I collagen and functions as a collagenase, we simultaneously visualized both MT1-MMP activity (using the AHLR biosensor) and collagenolysis (using rhodamine-conjugated collagen) at a single cell level. For this purpose, the MDA-MB-231 cells transfected with the AHLR biosensor were seeded on a cross-linked rhodamine-collagen film and cultured for 2 days [8], [10]. The ECFP/FRET ratio and collagen-rhodamine fluorescence images at the cell-matrix interface were recorded. As shown in Figure 6, both MT1-MMP activity and collagen degradation were clearly high in the MDA-MB-231 cells. There was also an increase of rhodamine fluorescence inside the cell bodies, probably due to the endocytosis of fragmented collagen [35]. As expected, GM6001 inhibited both MT1-MMP activity and collagen degradation in these cells (Fig. 6A). As a control, MT1-MMP-deficient SK-BR-3 cells did not cause detectable degradation of the rhodamine-conjugated collagen (data not shown). Taken together, these results suggest that our MT1-MMP FRET biosensor can serve as a powerful molecular tool for profiling the cellular MT1-MMP activity and assessing the invasive potential of single cells.

## Discussion

In breast cancer and many other cancer types, enzymatically active MT1-MMP resides at the extracellular surface of tumor and stromal cells. The proteolytic activity of MT1-MMP is considered critical for the tumor cells to modify extracellular matrix (ECM) and penetrate cross-linked human tissues [8], [10], [12], [36]. Consistently, MT1-MMP is highly expressed in aggressive breast and prostate tumor cells, and is associated with poor clinical outcomes [20]–[22]. In addition, MT1-MMP can be detected in the blood samples of cancer patients [23]. The mRNA level of MT1-MMP in peripheral blood has been shown to represent cancer progression [25], [37]. Therefore, the MT1-MMP activity of the tumor cells can serve as potential biomarker for cancer aggression. Accordingly, monitoring the levels of the cellular surface MT1-MMP activity is a key to the rational prediction of the invasion potential of cancer cells, which can provide a foundation for selecting treatments for cancer patients.

FRET-based biosensors can be genetically engineered and targeted to subcellular regions for monitoring the molecular activities in live cells with high spatiotemporal resolution [38]–[40]. The ability of the biosensors to report dynamic molecular activity at the single cell level provides an important means to evaluate the underlying cell-fate decision among a heterogeneous population [41]. In this work, we methodically optimized the linker length and the cleavage sequence of the MT1-MMP FRET biosensors by the *in vitro* cleavage assay. The AHLR biosensor with GGSGGT linkers displayed the best sensitivity to MT1-MMP proteolysis, suggesting a general approach to enhance biosensor sensitivity. The AHLR biosensor is also specific to MT1-MMP cleavage, albeit moderated less so in comparison to the AHLM and MHLR biosensors. More importantly, the AHLR biosensor cannot be cleaved by the anti-cancer MMP-8 *in vitro* (Figure S3). Although the AHLR biosensor with the GGS linker has been heuristically engineered and used in a previous publication from us[30], it has not been systematically characterized and optimized with other comparable biosensor constructs there. Overall, our selected AHLR biosensor is highly sensitive and sufficiently specific for assessing the MT1-MMP activity of individual cancer cells.

Although the invasive potential and MT1-MMP expression level of some of the tested cancer cell lines has been examined previously (Table S2), the enzymatic activity of MT1-MMP has not been directly measured or systematically studied to correlate with the invasiveness in the same cells. Here we successfully used the AHLR biosensor to differentiate invasive cancer cells from their non-invasive counterparts. Therefore, the reported MT1-MMP activity can be applied to profile and predict the invasiveness of new or unknown cancer cells. Furthermore, the MT1-MMP activity was significantly higher in adhesive cells than cells in suspension, suggesting that imaging technologies screening MT1-MMP activity in adhesive cells can be advantageous in detecting and assessing aggressive cancer cells.

Our results suggest that MT1-MMP activity is significantly up-regulated by cell-matrix interaction in the invasive cancer cells within 10 minutes of attachment. It remains unclear, however, how the MT1-MMP activity is up-regulated in these cells. It is possible that the ECM engagement of the hemopexin domain of MT1-MMP can inhibit the clathrin-dependent endocytosis of MT1-MMP and promote its stable localization at the plasma membrane [42]. The clustering and activation of integrin may also cause the dimerization and activation of membrane-tethered MT1-MMP [43]. As a result, the type and density of specific matrix proteins and the stiffness of matrix can all affect the detected MT1-MMP activity, which may constitute important future research directions. Interestingly, cells can easily adhere to the matrix independent of their MT1-MMP activity, suggesting that the MT1-MMP activity is a downstream signal dispensable for adhesion, although essential for invasion. When the SK-BR-3 cells were examined, their MT1-MMP activity and invasiveness cannot be activated by adhesion on fibronectin or collagen (data not shown). These results suggest that the molecular mechanism of distinct cell types in response to environmental ECM proteins can be different in determining their functional outcomes.

In summary, our work provides a powerful molecular tool for the detection of the MT1-MMP activity in single breast cancer cells, which should pave the road for profiling cancer invasiveness at single cell level and screening effective MT1-MMP inhibitors.

## Materials and Methods

### Gene Construction and DNA Plasmids

The original NL’ and the IV’ MT1-MMP biosensor were described previously [29], [44]. Briefly, The YPet cDNA was amplified by PCR with a sense primer containing a BglII site and a reverse primer containing a SacI site. ECFP was amplified by PCR with a sense primer containing a SacI site and the sequence of the MT1-MMP substrate peptide, and a reverse primer containing a PstI site, a stop codon, and a HindIII site. The substrate peptide sequences used for the MT1-MMP FRET biosensors were selected from a substrate phage library cleavable by MMPs including MT1-MMP, as well as an MT1-MMP cleavage site identified in proMMP-2 [29], [31].

The AHLR and RHLR biosensors were constructed by replacing the original NL substrate peptide (CPKESCNLFVLKD) in the NL’ biosensor with an optimized cleavage peptide sequences AHLR (CRPAHLRDSG) and RHLR (CRPRHLRDSG), respectively. These biosensors were further modified by PCR and ligation to insert GGS or GGSGGT linkers flanking the substrate sequence. The rest of the biosensors with GGSGGT linkers: the NL, HHLR, RHLM, RALR, MHLR, RGLR and AHLM biosensors, were constructed by replacing the substrate sequence with the corresponding substrate sequences. A negative control MT1-MMP biosensor was constructed by replacing the substrate cleavage site NL with a non-cleavable sequence IV.

The PCR products were fused together and cloned into pRSETb (Invitrogen) using BglII/HindIII sites for bacterial expression, and into pDisplay (Invitrogen) using BglII/PstI sites for mammalian cell expression. The pDisplay vector contains an N-terminal murine Ig κ–chain leader sequence, which directs the biosensor protein to the secretory pathway, and a C-terminal transmembrane domain of the platelet derived growth factor receptor β, which targets the biosensor protein to the plasma membrane.

### Protein expression, in vitro spectroscopy, and cleavage assays

The biosensor proteins were expressed with N-terminal 6×His tags in *Escherichia coli*, which were cultured for 16 h at 37°C and purified by nickel chelation chromatography as previously described [29]. The purified biosensor proteins were then mixed with the proteolysis assay buffer (50 mM HEPES, 10 mM CaCl_2_, 0.5 mM MgCl_2_, 50 µM ZnCl_2_, and 0.01% Brij-35, pH 6.8) at 37°C. For the *in vitro* assay, an MMP enzyme, such as the recombinant catalytic domains of human MT1-, MT2- or MT3-MMP (2 µg/ml, Mol. Wt. 20 kD, Calbiochem), or the active human MMP-2, MMP-8 or MMP-9 (6 µg/ml, Mol. Wt. 60 kD, Calbiochem), were then mixed with the biosensor-buffer solution (1 µM). The concentrations were adjusted to the molecular weight so that the catalytic domains and the full-length MMP have about the same molar concentration [29]. The ECFP/FRET emission ratio (476 nm/526 nm) of the biosensor was measured by a fluorescence plate reader (TECAN, Sapphire II) before and after mixing. The time courses of ECFP/FRET emission ratio were calculated from the recorded data in Excel (Microsoft), which were then compared among different groups after 2.5 hr of incubation. The biosensors tested include different substrate sequences flanked by the GGS or GGSGGT linker sequence as shown in Figure S2.

### Cell culture and Transfection

All cell lines were obtained from ATCC and cultured in the incubators at 37°C, supplemented with 95% air and 5% CO_2_. The MCF-7 cells were cultures in Eagle’s Minimum Essential Medium containing 0.01 mg/ml bovine insulin and 10% fetal bovine serum (FBS) as recommended by ATCC. The ZR-75-1 and CRL-2341 cells were cultured in the RPMI-1640 Medium and 10% FBS. The SK-BR-3 cells were cultured in McCoy’s 5a Medium Modified (ATCC) and 10% FBS. The CRL-4010 cells were cultured in the base medium for this cell line with additives from the MEGM kit (Lonza/Clonetics Corporation). The MDA-MB-231 cells were maintained in high glucose-DMEM (HyClone, Logan, UT) supplemented with 10% FBS (HyClone), 2 mM L-glutamine [45]. Different DNA plasmids were transfected into cells using Lipofectamine 2000 (Invitrogen) following the vendor protocol for 36–72 hours before imaging. Briefly, 0.5 µg/ml MT1-MMP biosensor DNA was used to perform lipofectamin transfection and 0.125 µg/ml MT1-MMP DNA was used for the co-transfection with the biosensor. The transfection efficiency was about 15–20%.

### Microscope Imaging

During imaging, the cells were maintained in CO_2_-independent medium (Gibco BRL) without serum at 37°C. Images were collected by a Zeiss Axiovert inverted microscope equipped with 100x objective (1.4 NA) and a cooled charge-coupled device camera (Cascade 512B; Photometrics) using the MetaFluor 6.2 software (Universal Imaging). The parameters of dichroic mirrors, excitation and emission filters for different fluorescence proteins were described previously [44]. In brief, the MT1-MMP biosensor was excited at the donor excitation wavelength of 420±20 nm, and the emissions collected at the donor emission wave length of 475±40 nm or the acceptor emission wave length of 535±25 nm for ECFP or FRET images, respectively. The excitation filter for ECFP at 420±20 nm was specifically selected to shift toward lower wavelength, away from the peak excitation spectrum of YPet to reduce the cross-excitation of the FRET acceptor YPet which has significantly higher brightness than ECFP [38]. This filter selection can minimize the effect of bleed-through on the FRET channel. The fluorescence intensity of non-transfected cells was quantified as the background signal and subtracted from the ECFP and FRET signals of transfected cells. The pixel-by-pixel ratio images of FRET/ECFP were calculated based on the background-subtracted fluorescence intensity images of ECFP and FRET by using the MetaFluor software. These ratio images were displayed in the intensity modified display (IMD) mode in which the color and brightness of each pixel is determined by the FRET/ECFP ratio and ECFP intensity, respectively. The emission ratio values were quantified and analyzed by Excel (Microsoft). Rhodamine was excited at 560±40 nm and the emission collected at 650±100 nm for collagen images.

### Inhibitor washout, adhesion assay, fibronectin and collagen-rhodamine coating

For inhibitor washout, cells were pre-incubated with 5 µM of GM6001 for 12 hours before being washed five times with HBSS [29]. The cells were then imaged to examine the effect of GM6001 removal and hence MT1-MMP activation.

For the adhesion assay, fibronectin coating (from bovine plasma, Sigma, diluted to 20 µg/ml) was prepared by incubation at 37°C for 4 hours. The MDA-MB-231 cells transfected with the biosensors were then plated on fibronectin-coated surface for subsequent imaging.

To prepare collagen-rhodamine coating [8], collagen stock (collagen type I rat tail in acetic acid 4.59 mg/ml, extracted without pepsin treatment, BD Biosciences) was diluted with borate buffer at pH 3 to a concentration of 1 mg/ml. The diluted collagen (100 µl) was mixed with PBS (200 µl, concentration 0.33 mg/ml) and used to coat the center well of the glass bottom dishes (Cell E&G) at 37°C for ∼3 hours. The collagen-coated dishes were then incubated with 2 ml borate buffer at pH 8.5 for 1 hour. 7 µl NHS-rhodamine solution (NHS-rhodamine 10 mg/ml in DMSO, Thermo Scientific) was dissolve in 300 µl borate buffer at pH 8.5 and applied to the collagen-coated dishes for 2-3 hours to allow conjugation between the surface of collagen gel and rhodamine. The dishes were subsequently washed with PBS 5 times and incubated with PBS before passing cells on them.

## Supporting Information

Figure S1 **The design principle of MT1-MMP FRET biosensors. (A)** The schematics of the MT1-MMP biosensors. **(B)** The activation mechanism of the MT1-MMP biosensor.
(TIF)

Figure S2 **The design principle of the three most sensitive MT1-MMP biosensors with the optimal GGSGGT linker.** **(A)** The schematic drawing of the optimized MT1-MMP biosensors, each containing a cyan fluorescence protein (ECFP), a substrate peptide (AHLR, AHLM, or MHLR) flanked by GGSGGT linkers at both sides, and a yellow fluorescence protein (YPet). **(B)** The activation mechanism of the membrane-tagged MT1-MMP biosensor expressed at the extracellular surface of the plasma membrane in a live cell.
(TIF)

Figure S3 **Characterize the specificity of the AHLR biosensor against anti-cancer MMP-8.** The time course of the biosensor ECFP/FRET emission ratio is compared upon the incubation with the active catalytic domain of MT1-MMP or the full-length MMP-8.
(TIF)

Figure S4 **Profile the invasive MT1-MMP activity in breast cancer cell lines utilizing GM6001 washout assay.** For breast cancer cell lines **(A)** CRL 2314 and **(B)** ZR-75-1, the ECFP/FRET emission ratio images are shown for a representative cell before (left) and after (middle) GM6001 washout. The time course of the quantified ECFP/FRET ratio value normalized to its average value before GM6001 washout is plotted in the right panel.
(TIF)

Table S1 **The sequences of the most sensitive and specific cleavage substrate of MT1-MMP.** The sequences shown in the rows were identified using the cleavage of the substrate phage libraries (10×10^9^ individual phages each expressing a random hexamer peptide sequence) by the individual purified MMPs, including MT1-MMP. The residues shown at the positions P5, P4 and P3’-P5’ are equally favorable for MT1-MMP proteolysis. The columns P1-P1’ show the amino acids recognized as the cleavage site. In the score column, a high score indicates a high sensitivity and specificity of the sequence being preferably cleaved by MT1-MMP comparing to other MMPs.
(TIF)

Table S2 **The MT1-MMP expression level and the invasiveness of the cancer cell lines.** HeLa is an immortal cell line derived from a glandular cancer of the cervix [46], which expresses little MT1-MMP protein and could not invade across collagen. So HeLa cells were chosen as the negative control, while those overexpressing exogenous MT1-MMP as the positive control. The fibrosarcoma-derived HT1080 cells and the breast cancer cells MDA-MB-231 were found to be highly invasive in collagen gel and express high levels of MT1-MMP [47]-[49]. In comparison to the MDA-MB-231 cells, the low invasive breast cancer cells MCF-7 and ZR-75-1 cells have been shown to express low levels of MT1-MMP [50]-[52]. The breast cancer cells (SK-BR-3) have low invasiveness, but were reported with contradictive MT1-MMP expressions [51], [53]-[55]. The Her2 negative breast cancer cells CRL2314 (ATCC HCC-48) have a low level of MT1-MMP expression, but their invasiveness is relatively unknown [56]. The MT1-MMP expression or invasive potential of the immortal breast epithelial cells CRL4010 (ATCC CRL_4010) is not clear.
(TIF)

Movie S1 **MT1-MMP activation during the inhibitor wash-out assay.** A live MDA-MB-231 cell was transfected with the AHLR biosensor and pre-incubated with a MMP inhibitor, GM6001. The cell was monitored while the inhibitor was washed out from the medium. **Left panel:** the video sequence of the DIC images; **right panel:** the video sequence of the ECFP/FRET ratio images of the same cell changing color from blue to red, representing the activation of MT1-MMP.
(AVI)

Movie S2 **MT1-MMP activation during cell adhesion.** A live MDA-MB-231 cell expressing the AHLR biosensor was dropped at the surface of cover glass coated with fibronectin, to allow the cell to adhere and spread. The cell was monitored during the adhesion process (center of frame). The ECFP/FRET ratio at the cell peripheral turned red as the cell expanded, representing the activation of MT1-MMP. **Left panel:** the video sequence of DIC images; **right panel:** the video sequence of ECFP/FRET ratio images of the same cell.
(AVI)
