# Supplementary figures and images for: Quantitative FRET Imaging to Visualize the Invasiveness of Live Breast Cancer Cells
Source: PLoS One. 2013 Mar 13;8(3):e58569. doi: 10.1371/journal.pone.0058569 (PMC3596289; doi:10.1371/journal.pone.0058569)

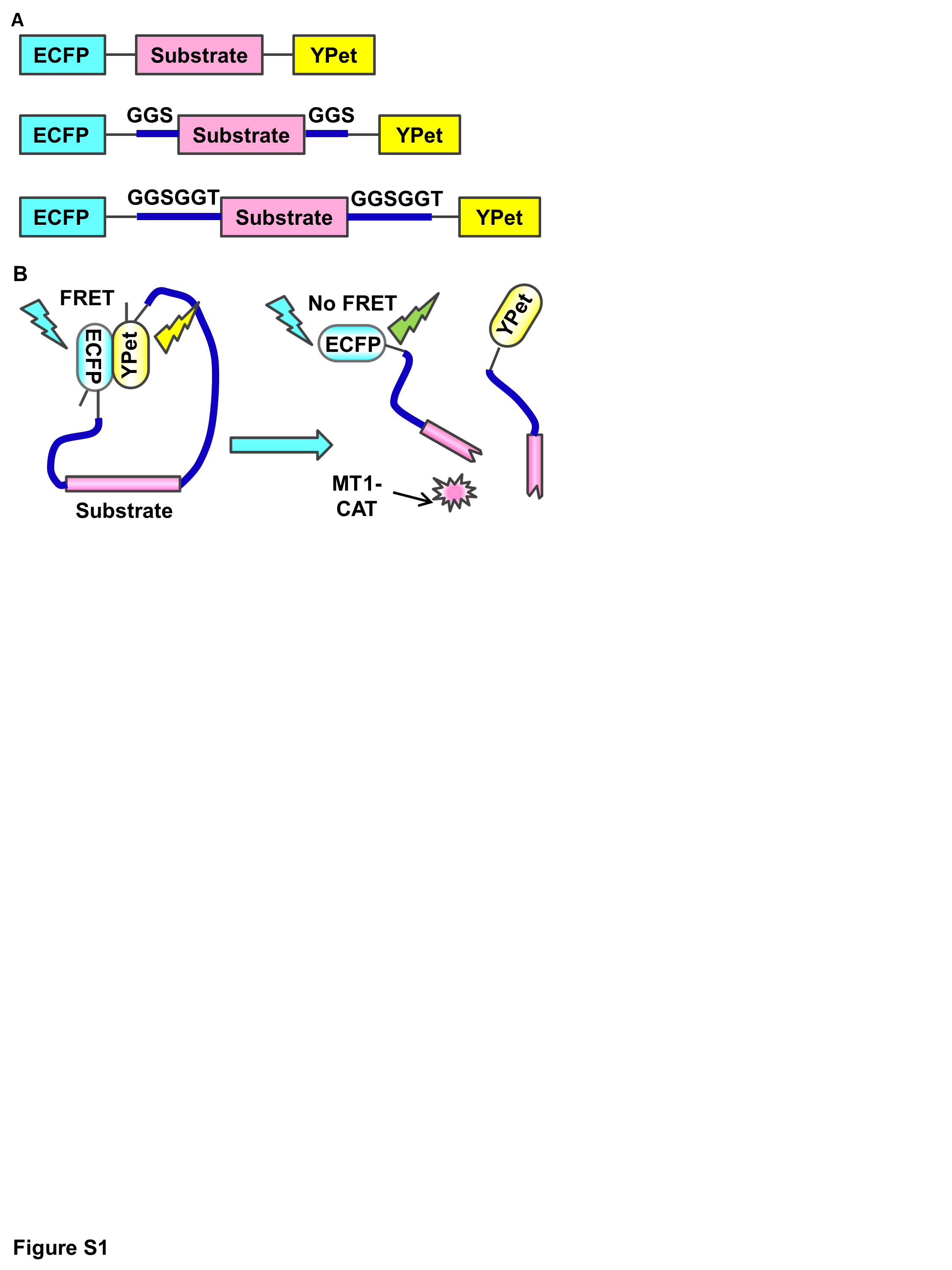

Supplement: Figure S1 — The design principle of MT1-MMP FRET biosensors. (A) The schematics of the MT1-MMP biosensors. (B) The activation mechanism of the MT1-MMP biosensor. (TIF) [file pone.0058569.s001.tif]

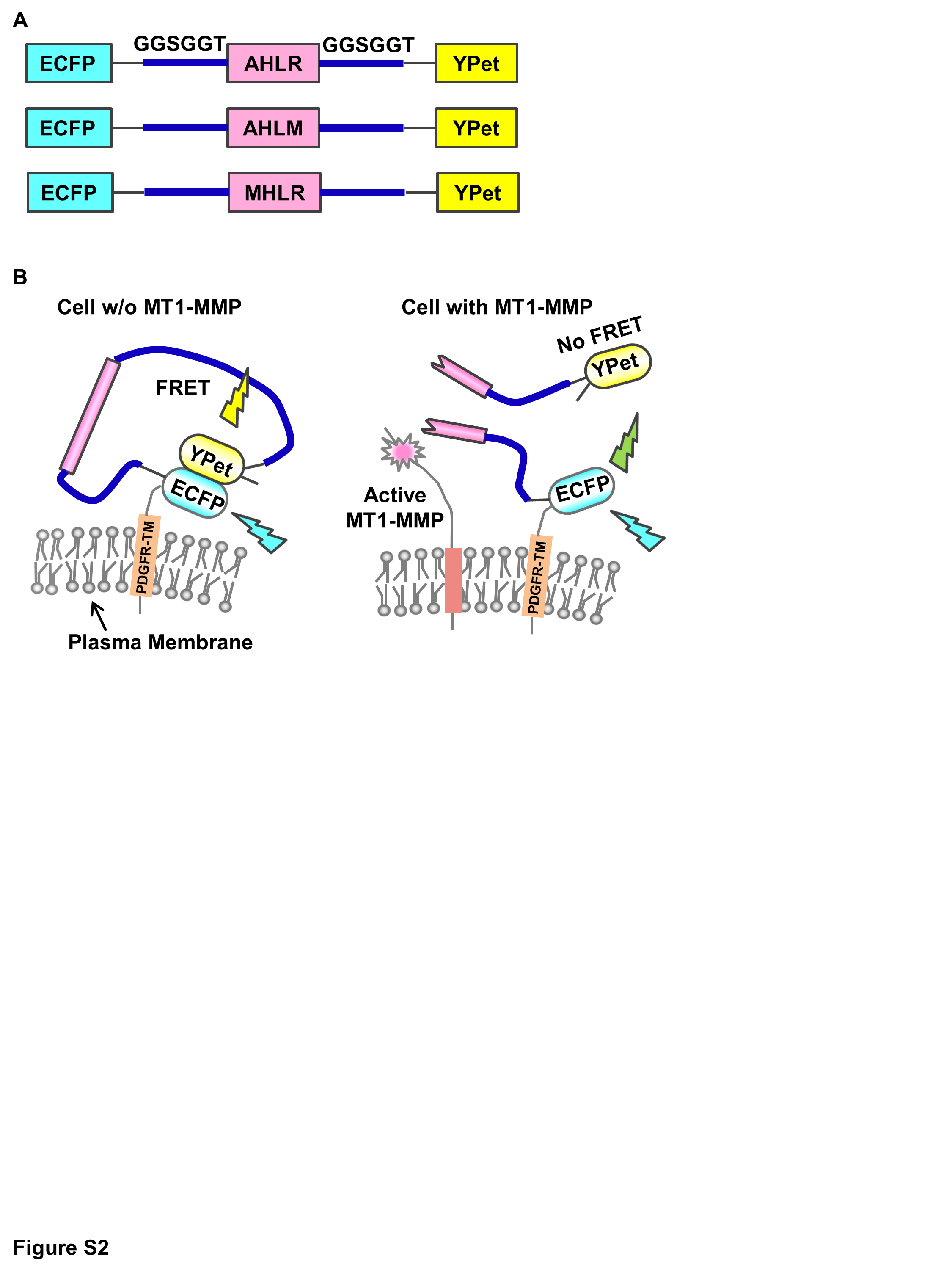

Supplement: Figure S2 — The design principle of the three most sensitive MT1-MMP biosensors with the optimal GGSGGT linker. (A) The schematic drawing of the optimized MT1-MMP biosensors, each containing a cyan fluorescence protein (ECFP), a substrate peptide (AHLR, AHLM, or MHLR) flanked by GGSGGT linkers at both sides, and a yellow fluorescence protein (YPet). (B) The activation mechanism of the membrane-tagged MT1-MMP biosensor expressed at the extracellular surface of the plasma membrane in a live cell. (TIF) [file pone.0058569.s002.tif]

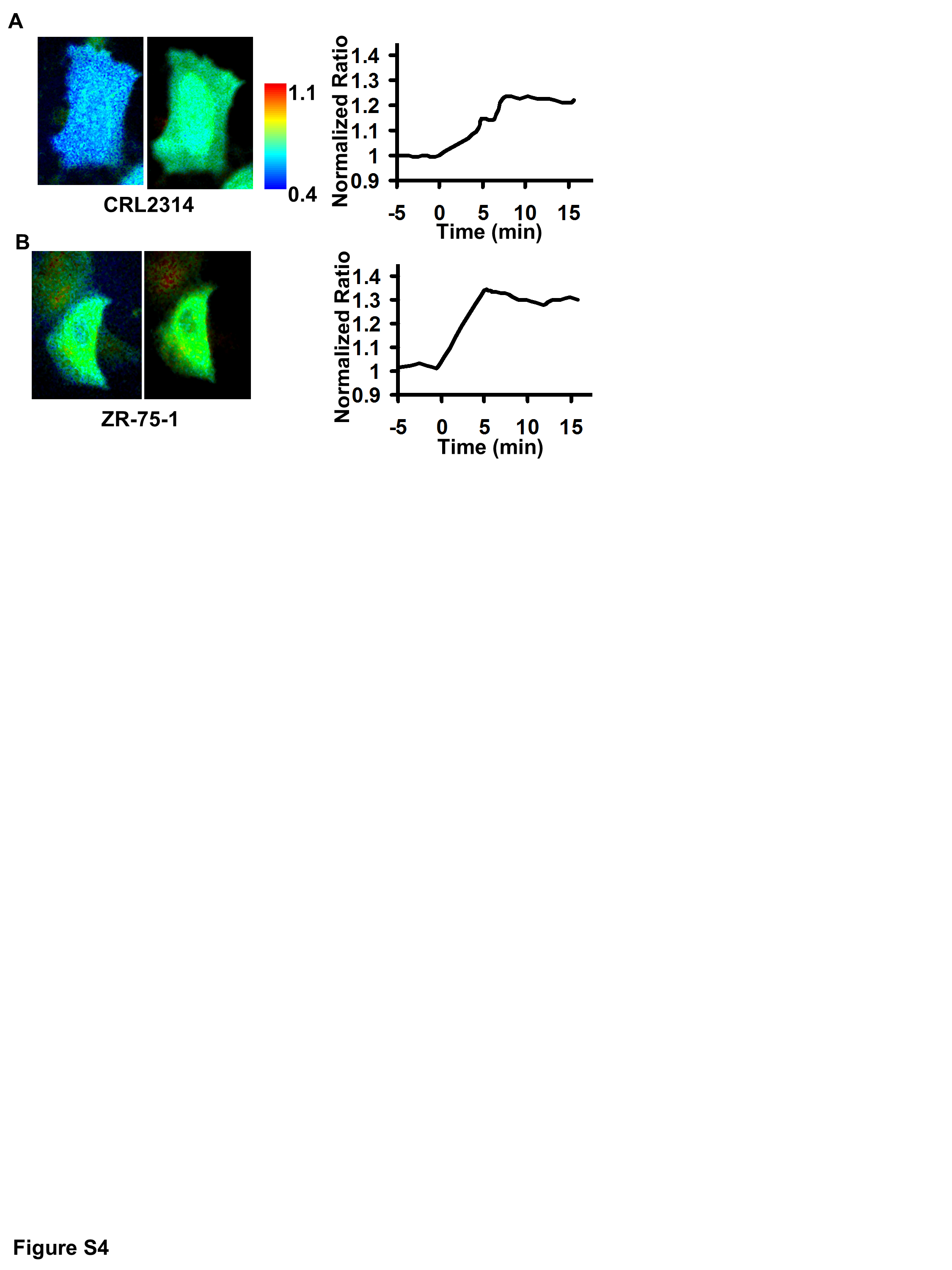

Supplement: Figure S4 — Profile the invasive MT1-MMP activity in breast cancer cell lines utilizing GM6001 washout assay. For breast cancer cell lines (A) CRL 2314 and (B) ZR-75-1, the ECFP/FRET emission ratio images are shown for a representative cell before (left) and after (middle) GM6001 washout. The time course of the quantified ECFP/FRET ratio value normalized to its average value before GM6001 washout is plotted in the right panel. (TIF) [file pone.0058569.s004.tif]

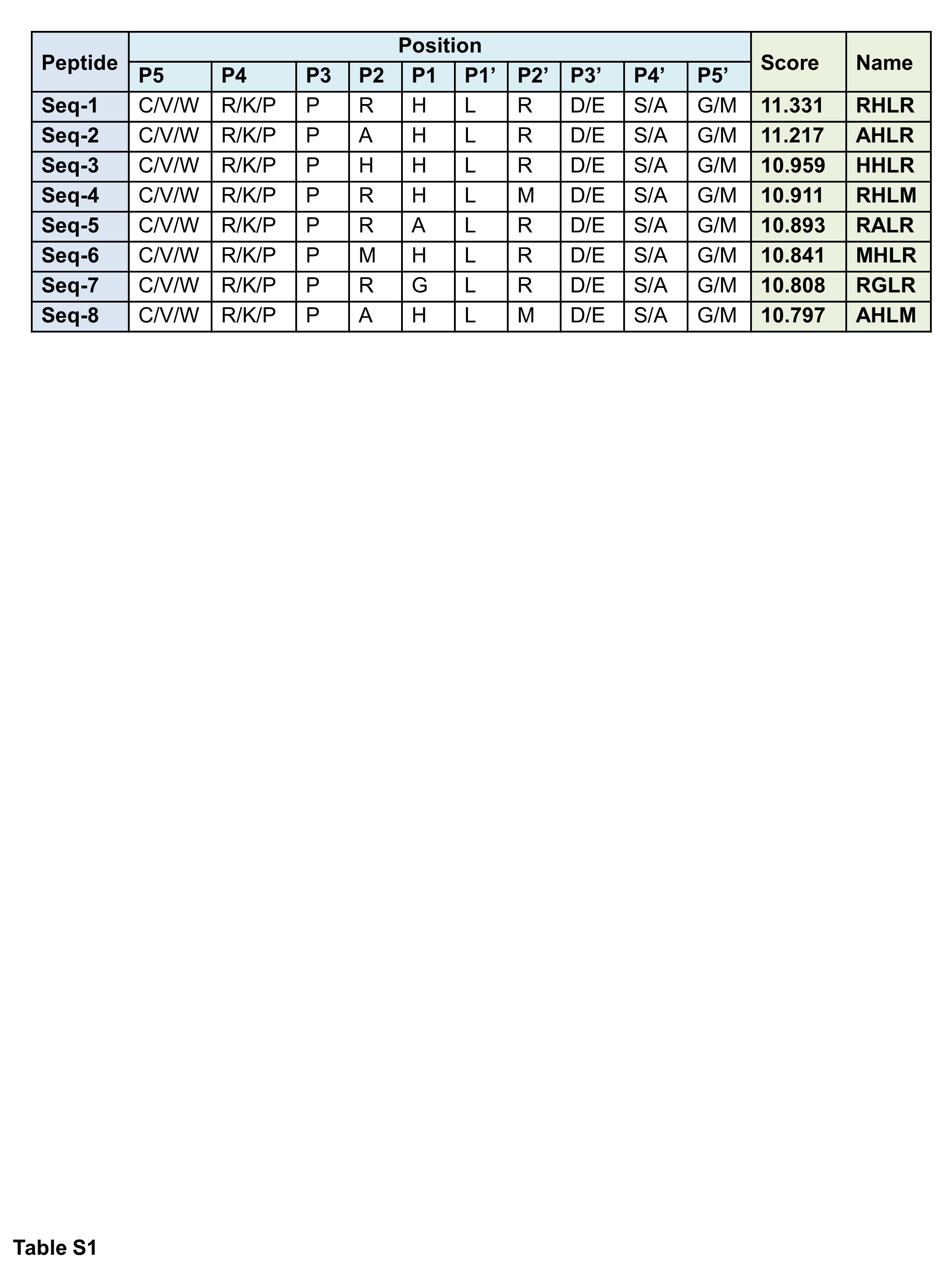

Supplement: Table S1 — The sequences of the most sensitive and specific cleavage substrate of MT1-MMP. The sequences shown in the rows were identified using the cleavage of the substrate phage libraries (10×109 individual phages each expressing a random hexamer peptide sequence) by the individual purified MMPs, including MT1-MMP. The residues shown at the positions P5, P4 and P3’-P5’ are equally favorable for MT1-MMP proteolysis. The columns P1-P1’ show the amino acids recognized as the cleavage site. In the score column, a high score indicates a high sensitivity and specificity of the sequence being preferably cleaved by MT1-MMP comparing to other MMPs. (TIF) [file pone.0058569.s005.tif]
